# Supplementary material for: A network model of path control
Source: bioRxiv. 2026 Oct 9:2026.10.02.756268. Preprint. [Version 1] doi: 10.64898/2026.10.02.756268 (PMC13692032; doi:10.64898/2026.10.02.756268)
Supplement: Supplementary file 1 [file NIHPP2026.10.02.756268V1-supplement-1.pdf]

bioRxiv preprint doi: https://doi.org/10.64898/2026.10.02.756268; this version posted October 9, 2026. The copyright holder for this preprint (which was not certified by peer review) is the author/funder, who has granted bioRxiv a license to display the preprint in perpetuity. It is made available under a CC-BY 4.0 International license.

Appendix

A Dynamics of the Fourier coefficients

In this section, we derive the governing equations for the Fourier coefficients (first harmonic and mean) of the activity profiles. The model in the main text is an example of a spatially organized network with multiple cell types and translationally invariant connections [62–64]. This connectivity is organized around an angular variable, more specifically making the model a ring network [29, 36, 65, 66]. In the linear case, the dynamics of such systems can be expressed as follows:

$$\tau \frac{\partial}{\partial t} x_j(\theta, t) = -x_j(\theta, t) + \sum_{k=1}^{N} \int_0^{2\pi} d\theta' w_{jk}(\theta - \theta') x_k(\theta', t) + b_j v(\theta, t). \tag{11}$$

Here, $v(\theta, t)$ is some external input and $w_{jk}(\cdot)$ is a connectivity kernel (periodic over 2π) that relates the strength of a connection to the cell types and relative positions of the two neurons. For simplicity, we model the network as a continuum, though translating to the discrete case is straightforward (see [29]).

As is standard in the study of such networks, we can use the Fourier transform to express the shape of each population's activity profile in the frequency domain. Let $\hat{z}_j^{(\ell)} \in \mathbb{C}$ denote the ℓth (with $\ell \in \mathbb{N}$) unnormalized Fourier coefficient of the jth activity profile:

$$\hat{z}_j^{(\ell)}(t) = \int_0^{2\pi} d\theta \, e^{i\ell\theta} x_j(\theta, t), \tag{12}$$

where $i = \sqrt{-1}$ denotes the imaginary unit (used elsewhere in this manuscript as a discrete indexing variable). Similarly, let $\hat{w}_{jk}^{(\ell)}$ and $\hat{v}^{(\ell)}(t)$ denote the ℓth Fourier coefficients of the connection kernels and input pattern. The dynamics of $\hat{z}_j^{(\ell)}$ follow

$$\tau \frac{d}{dt} \hat{z}_j^{(\ell)}(t) = -\hat{z}_j^{(\ell)}(t) + \sum_{k=1}^{N} \hat{w}_{jk}^{(\ell)} \hat{z}_k^{(\ell)}(t) + b_j \hat{v}^{(\ell)}(t). \tag{13}$$

Note that $\hat{z}_j^{(\ell)}(t)$ interacts with the ℓth Fourier coefficients of the other activity profiles and the input but is completely uncoupled from Fourier coefficients corresponding to other frequencies ($m \neq \ell$). Each Fourier mode thus defines an independent dynamical system driven by the corresponding Fourier coefficient of the input and coupled through the corresponding Fourier coefficients of the connectivity kernels:

$$\tau \frac{d}{dt} \hat{\mathbf{z}}^{(\ell)}(t) = -\hat{\mathbf{z}}^{(\ell)}(t) + \hat{\mathbf{W}}^{(\ell)} \hat{\mathbf{z}}^{(\ell)}(t) + \hat{v}^{(\ell)}(t) \mathbf{b}, \tag{14}$$

where $\hat{\mathbf{z}}^{(\ell)}(t) \in \mathbb{C}^N$ is the population vector with entries $\hat{z}_j^{(\ell)}$ and $\hat{\mathbf{W}}^{(\ell)} \in \mathbb{C}^{N \times N}$ is the coupling matrix with entries $\hat{w}_{jk}^{(\ell)}$ (see [29] and [64] for analogous modal reductions).

Because the external input in the main text is a simple sinusoid lacking any high frequency content ($\hat{v}^{(\ell)}(t) = 0$ for all $\ell > 1$), its activity profiles will not exhibit high frequency content unless it is present at initialization. In addition, assuming a given mode's dynamics are stable ($\operatorname{Re} \lambda_j \left(\hat{\mathbf{W}}^{(\ell)} \right) < 1$ for all j), any high frequency content present at initialization will dissipate. In any case, we assume an initial state of zero ($x_j(\theta, 0) = 0$) throughout, thus guaranteeing that the activity profiles are always simple sinusoids and reducing the dynamics to modes 0 and 1. In the main text, for clarity, we expressed these modes using real (rather than complex) numbers and rescaled them so that their magnitudes directly equal the associated features (mean and amplitude) of the activity profile:

$$\mu_j(t) = \frac{1}{2\pi} \hat{z}_j^{(0)}(t) \in \mathbb{R}, \tag{15}$$

$$\hat{\mathbf{x}}_j^{(1)}(t) = \frac{1}{\pi} \begin{bmatrix} \operatorname{Re} \hat{z}_j^{(1)}(t) \\ \operatorname{Im} \hat{z}_j^{(1)}(t) \end{bmatrix} \in \mathbb{R}^2. \tag{16}$$

bioRxiv preprint doi: https://doi.org/10.64898/2026.10.02.756268; this version posted October 9, 2026. The copyright holder for this preprint (which was not certified by peer review) is the author/funder, who has granted bioRxiv a license to display the preprint in perpetuity. It is made available under a CC-BY 4.0 International license.

Finally, to obtain the governing equations for these variables, we consider the specific connectivity kernel used in the main text. This connectivity kernel is a weighted sum of two delta functions, respectively corresponding to the phase-preserving and phase-inverting connections:

$$w_{jk}(\theta) = W_{jk}^{+}\delta_{2\pi}(\theta) + W_{jk}^{-}\delta_{2\pi}(\theta - \pi). \tag{17}$$

The Fourier coefficients of this kernel depend on the parity of the mode:

$$\hat{w}_{jk}^{(\ell)} = W_{jk}^{+} + (-1)^{\ell} W_{jk}^{-}.$$

This parity dependence reflects the fact that a phase shift of π leaves modes of even wavenumber unchanged while multiplying modes of odd wavenumber by -1. Consequently, phase-inverting connections act as negative, "effectively inhibitory" couplings among the first (and other odd-numbered) harmonics of the activity profiles, even though all the units in the network are excitatory.

B Dynamics of the mean

The activity of each neural array in our model can be decomposed into two components: the first harmonic and the mean. In this section, we consider the dynamics of the latter. Let $\boldsymbol{\mu} \in \mathbb{R}^N$ denote the mean activity in each array. The dynamics of $\boldsymbol{\mu}$ follow

$$\tau \frac{d}{dt}\boldsymbol{\mu}(t) = -\boldsymbol{\mu}(t) + \left(\mathbf{W}^{+} + \mathbf{W}^{-}\right)\boldsymbol{\mu}(t) + \mathbf{b}\|\mathbf{v}(t)\|. \tag{18}$$

Whereas the first harmonic of the network's input encodes the agent's velocity vector, the mean of the input encodes the agent's translational speed (due to the non-zero offset in equation 1). As a result, whereas the first harmonic computes a displacement-like quantity by temporally filtering its velocity vector input, the mean computes a path-length-like quantity by temporally filtering its speed input. In addition, note that phase-inverting connections positively couple the means but negatively couple the first harmonics. Due to this coupling difference, the two components will generally apply different filters to their respective inputs when the network contains phase-inverting connections.

Finally, since the network's connections and input are non-negative, single-neuron activity must also be non-negative. As a result, the mean of each array's activity profile must always equal or exceed the amplitude of its first harmonic. Since the mean bounds the magnitude of the first harmonic, the stability of the network solely depends on the stability of the mean dynamics, with stability condition $\lambda_{\max}(\mathbf{W}^{+} + \mathbf{W}^{-}) < 1$.

C Linear filtering

Many of the results in the main text rely on the fact that time-invariant linear dynamical systems act as temporal filters of their inputs. In this section, we review the relevant mathematics, which constitute standard linear systems theory (see e.g. [25, 26]). Consider a system of the form

$$\tau \frac{d}{dt}\mathbf{x}(t) = \mathbf{A}\mathbf{x}(t) + u(t)\mathbf{b}, \tag{19}$$

$$y(t) = \mathbf{c}^{\top}\mathbf{x}(t). \tag{20}$$

Here $\mathbf{x}(t) \in \mathbb{R}^N$ is the system state, $u(t) \in \mathbb{R}$ is some time-varying input to the system, $y(t) \in \mathbb{R}$ is some observed variable, $\mathbf{A} \in \mathbb{R}^{N\times N}$ is a matrix of recurrent couplings, $\mathbf{b} \in \mathbb{R}^N$ is a vector of input weights, and $\mathbf{c} \in \mathbb{R}^N$ is a vector of the readout weights. Again, we will assume an initial condition of $\mathbf{x}(0) = \mathbf{0}$.

We can express $y(t)$ as some convolution of $u(t)$ with a kernel that depends on the system's coupling parameters:

$$y(t) = (\mathcal{H}[\mathbf{A},\mathbf{b},\mathbf{c},\tau] * u)(t) = \int_0^t dt'\, \mathcal{H}[\mathbf{A},\mathbf{b},\mathbf{c},\tau](t-t')u(t').$$

bioRxiv preprint doi: https://doi.org/10.64898/2026.10.02.756268; this version posted October 9, 2026. The copyright holder for this preprint (which was not certified by peer review) is the author/funder, who has granted bioRxiv a license to display the preprint in perpetuity. It is made available under a CC-BY 4.0 International license.

Here, $\mathcal{H}$ is a functional that takes in the system parameters and returns the associated temporal kernel:

$$\mathcal{H}[\mathbf{A}, \mathbf{b}, \mathbf{c}, \tau](t) = \mathbb{1}\{t > 0\} \cdot \frac{1}{\tau}\mathbf{c}^\top \exp\left(\frac{t}{\tau}\mathbf{A}\right)\mathbf{b}, \tag{21}$$

where the coefficient $\mathbb{1}\{t > 0\}$ ensures that the kernel is causal (equals 0 for $t < 0$). In general, such a filter can be expressed as a sum of up to N terms, each consisting of a polynomial times an exponential. Thus, for $t > 0$:

$$\mathcal{H}[\mathbf{A}, \mathbf{b}, \mathbf{c}, \tau](t) = \frac{1}{\tau}\sum_i P_i\left(\frac{t}{\tau}\right)\exp\left(\lambda_i(\mathbf{A})\frac{t}{\tau}\right), \tag{22}$$

where $P_i\left(\frac{t}{\tau}\right)$ is a polynomial function of time and $\lambda_i(\mathbf{A}) \in \mathbb{C}$ denotes the ith eigenvalue of $\mathbf{A}$.

If the matrix $\mathbf{A}$ is diagonalizable, then each $P_i\left(\frac{t}{\tau}\right) = p_i$ will be a constant and the filter will simply be a weighted sum of exponentials. Non-diagonalizable systems with nontrivial polynomial terms can exhibit interesting filtering properties that have been explored elsewhere [28, 67].

The behavior of exponential terms will in turn depend on the eigenvalues of $\mathbf{A}$. Real-valued eigenvalues will contribute exponential decay terms that (leakily) integrate the input with an effective time constant of $\tau_i = -\frac{\tau}{\operatorname{Re}\lambda_i(\mathbf{A})}$. Complex-valued modes will come in conjugate pairs, resulting in exponentially damped (or driven) oscillatory terms of the form

$$\exp\left(-\frac{t}{\tau_i}\right)\cos\left(2\pi\omega_i\frac{t}{\tau} + \phi_i\right), \tag{23}$$

where $\omega_i \in \mathbb{R}$ determines the oscillation's frequency and $\phi_i \in [0, 2\pi)$ determines its phase. These oscillatory terms can realize more complex (e.g. band-pass) filtering properties.

With this background in mind, we can summarize many of the variables in the main text as temporally filtered responses to external inputs:

Variable	Filter	Input
$\hat{\mathbf{x}}_i^{(1)}(t)$	$\mathcal{H}[\hat{\mathbf{W}}^{(1)} - \mathbf{I}, \mathbf{b}, \mathbf{e}_i, \tau]$	$\mathbf{v}(t)$
$\mu_i(t)$	$\mathcal{H}[\mathbf{W}^+ + \mathbf{W}^- - \mathbf{I}, \mathbf{b}, \mathbf{e}_i, \tau]$	$\lVert\mathbf{v}(t)\rVert$
$k_U\hat{\mathbf{u}}^{(1)}(t)$	$\mathcal{H}[\hat{\mathbf{W}}^{(1)} - \mathbf{I}, \mathbf{b}, \mathbf{c}, \tau]$	$\mathbf{v}(t)$
$\mathbf{v}(t)$	$\mathcal{H}[\mathbf{A}, \mathbf{e}_1, \mathbf{e}_1, \tau]$	$\mathbf{f}(t)$

Above, $\mathbf{e}_i$ denotes the ith standard basis vector (the vector with entries δ_{ij}).

D Polar coordinate description

In this section, we reexpress the dynamics of the closed-loop system in polar coordinates. This change of coordinates yields governing equations for the agent's heading and forward speed, thereby making connections to the literature on central complex output circuits more apparent. It also yields governing equations for the activity profile phases and amplitudes, thereby providing a different perspective on the neural dynamics.

To start, we will define polar representations for each of the variables. Let $\hat{\mathbf{x}}_i^{(1)} \leftrightarrow (X_i, \theta_i)$ where X_i denotes the amplitude of the ith activity profile and θ_i denotes its phase; $\hat{\mathbf{u}}^{(1)} \leftrightarrow (U, \psi)$, where U denotes the amplitude of the setpoint signal and ψ denotes its phase; and $\mathbf{v} \leftrightarrow (V, \phi)$, where V denotes the agent's forward speed and ϕ denotes its heading. Note that "heading" simply refers to the direction of the velocity vector in this model. Since the agent is modeled as a particle without an intrinsic axis of orientation, the model does not distinguish between heading and direction of travel.

bioRxiv preprint doi: https://doi.org/10.64898/2026.10.02.756268; this version posted October 9, 2026. The copyright holder for this preprint (which was not certified by peer review) is the author/funder, who has granted bioRxiv a license to display the preprint in perpetuity. It is made available under a CC-BY 4.0 International license.

When equation 7 is rewritten in polar coordinates, the resulting heading dynamics recapitulate the basic features of the steering control loop implemented by PFL3 cells [6, 15, 19]:

$$\begin{aligned}\tau V(t)\frac{d}{dt}\phi(t) &= -k_U U(t)\sin\left(\phi(t)-\psi(t)\right)+f_\phi(t)\\ &= -\sum_{i=1}^{N} c_i X_i(t)\sin\left(\phi(t)-\theta_i(t)\right)+f_\phi(t).\end{aligned} \tag{24}$$

This equation continuously compares the agent's current heading $\phi(t)$ to the setpoint phase $\psi(t)$, yielding an "alignment error" $\sin(\phi(t)-\psi(t))$. The resulting heading update is then proportional to the negative error, which causes the agent to turn towards the setpoint phase. In our model, the gain of this turning drive scales with $U(t)$, the amplitude of the setpoint signal. Additionally, as the second line of equation 24 shows, we can also decompose the full turning output into a weighted sum of analogous contributions from the N arrays. From this latter perspective, each array steers the agent's heading towards its activity phase $\theta_j(t)$. The strength of this pull depends on the amplitude $X_j(t)$ and the output weight c_j. In our model, turning gain is also inversely proportional to forward speed $V(t)$. Finally, $f_\phi(t) \in \mathbb{R}$ denotes the effect of the forcing function orthogonal to the current heading.

The radial component of the polar dynamics relates the agent's forward speed to the degree of alignment between the heading and setpoint, as well as the amplitude of the setpoint signal. There are populations of output neurons—namely, PFL2 cells [19]—well-poised to implement such a speed controller. More formally,

$$\begin{aligned}\tau\frac{d}{dt}V(t) &= -\gamma V(t)+k_U U(t)\cos(\phi(t)-\psi(t))+f_V(t)\\ &= -\gamma V(t)+\sum_{i=1}^{N} c_i X_i(t)\cos(\phi(t)-\theta_i(t))+f_V(t).\end{aligned} \tag{25}$$

This equation consists of a drag-like term that drives forward speed to 0 in the absence of input. The neural drive that combats this tendency is proportional to $\cos(\psi(t)-\phi(t)) \in [-1,1]$, which quantifies the degree of alignment between the setpoint signal and heading. Ignoring the forcing function and treating the other state variables as fixed, this equation will drive forward speed to a maximal value $\frac{k_U U(t)}{\gamma}$ when heading and setpoint are perfectly aligned, a speed of 0 when the two are orthogonal, and a speed of $-\frac{k_U U(t)}{\gamma}$ when the two are oppositely oriented. We can view moments of transition from positive to negative V as instantaneous inversions of the heading rather than backwards movement, since the points $(-V,\phi)$ and $(+V,\phi+\pi)$ are mathematically equivalent in polar coordinates. Like the turning dynamics, the forward speed dynamics can also be decomposed into a linear combination of analogous per-array contributions (second line of equation 25). In addition, $f_V(t) \in \mathbb{R}$ denotes the effect of the forcing function aligned with the current heading.

Finally, to understand the joint dynamics of neural activity and behavior, we rewrite the neural dynamics along the first harmonic in polar coordinates. The phase dynamics are given by

$$\tau X_i(t)\frac{d}{dt}\theta_i(t) = -\sum_{j=1}^{N}(W_{ij}^{+}-W_{ij}^{-})X_j(t)\sin(\theta_i(t)-\theta_j(t)) - b_i V(t)\sin(\theta_i(t)-\phi(t)), \tag{26}$$

which is analogous to the governing equation that relates neural activity to changes in heading. Similarly, the amplitude dynamics are given by

$$\tau\frac{d}{dt}X_i(t) = -X_i(t)+\sum_{j=1}^{N}(W_{ij}^{+}-W_{ij}^{-})X_j(t)\cos(\theta_i(t)-\theta_j(t)) + b_i V(t)\cos(\theta_i(t)-\phi(t)), \tag{27}$$

which is analogous to the equation relating neural activity to changes in forward speed. In summary, the phases of the neural activity bumps and the agent's heading define a set of angular variables coupled by alignment (and amplitude-dependent) interactions, somewhat akin to the synchronizing forces in a Kuramoto model [68].

bioRxiv preprint doi: https://doi.org/10.64898/2026.10.02.756268; this version posted October 9, 2026. The copyright holder for this preprint (which was not certified by peer review) is the author/funder, who has granted bioRxiv a license to display the preprint in perpetuity. It is made available under a CC-BY 4.0 International license.

E Statistical structure of random walks

To illustrate how the network's couplings can shape the agent's trajectories through its environment, we model the forcing function as a minimally structured source of random variability:

$$\mathbf{f}(t) = \sigma \boldsymbol{\xi}(t), \tag{28}$$

where $\boldsymbol{\xi}(t) \in \mathbb{R}^2$ is a white noise process with $\langle \boldsymbol{\xi}(t) \rangle = 0$ and $\langle \boldsymbol{\xi}(t)\boldsymbol{\xi}(t')^\top \rangle = \delta(t-t')\mathbf{I}$, where $\langle \cdot \rangle$ denotes an expectation over noise realizations. In addition, $\sigma \in \mathbb{R}_+$ is a constant that sets the noise strength. With this stochastic forcing, the agent will perform a correlated random walk through its environment [30, 31].

The overall trajectory will reflect a weighted sum of the $N+1$ modes present in the joint system. For concreteness, we will assume the closed-loop coupling matrix $\mathbf{A}$ is diagonalizable and its eigenvalues are real, so that the temporal filter realized by the agent's velocity is a sum of exponentials of the form

$$\mathcal{H}\left[\mathbf{A}, \mathbf{e}_1, \mathbf{e}_1, \tau\right](t) = \frac{1}{\tau} \sum_{i=1}^{N+1} a_i \exp\left(-\frac{t}{\tau_i}\right), \tag{29}$$

where $a_i \in \mathbb{R}$ and $\tau_i = -\frac{\tau}{\lambda_i(\mathbf{A})} \in \mathbb{R}$ are the coefficient and effective time constant associated with the ith mode of the filter. We further assume that the joint system is stable ($\lambda_i(\mathbf{A}) < 0$ for all i).

With such a filter, the random walk will exhibit a multiplicity of time scales that are evident in its autocovariance. The autocovariance relates the expected similarity (inner product) of the velocity vector at two time points to the duration $\Delta \geq 0$ separating them. In this case, the autocovariance of the agent's random walk will contain up to $N+1$ modes that are inherited from the filter:

$$C(\Delta) = \lim_{t\to\infty} \langle \mathbf{v}(t)^\top \mathbf{v}(t+\Delta) \rangle = \frac{2\sigma^2}{\tau^2} \sum_{i=1}^{N+1} \left(\sum_{j=1}^{N+1} \frac{a_j \tau_j}{\tau_i + \tau_j} \right) a_i \tau_i \exp\left(-\frac{\Delta}{\tau_i}\right). \tag{30}$$

Each term in this weighted sum contributes a correlation time scale of τ_i. Terms with positive coefficients promote persistent movement over lags $\Delta < \tau_i$, while terms with negative coefficients promote reversals over the same time scale. The overall path structure will reflect the combined contributions of each mode.

The filter's time scales are also present in the agent's mean squared displacement (MSD), which characterizes how the agent's distance from the starting point of an interval scales with the interval's duration:

$$\mathrm{MSD}(\Delta) = \lim_{t\to\infty} \left\langle \left\| \int_t^{t+\Delta} dt'\, \mathbf{v}(t') \right\|^2 \right\rangle = \frac{4\sigma^2}{\tau^2} \sum_{i=1}^{N+1} \left(\sum_{j=1}^{N+1} \frac{a_j \tau_j}{\tau_i + \tau_j} \right) a_i \tau_i^2 \left(\Delta - \tau_i \left(1 - \exp\left(-\frac{\Delta}{\tau_i}\right)\right)\right). \tag{31}$$

Finally, if the joint system has complex modes, the filter will contain exponentially damped oscillatory terms. Such modes will encourage periodic backtracking in the agent's trajectory and contribute oscillatory terms with the same frequency and decay rate to the autocovariance.

F Navigation towards an intermittent signal

To model navigation towards an intermittent sensory cue, we augment the forcing function with a temporally intermittent signal—mathematically equivalent to "shot noise" [31, 69]—that biases the agent towards some cue direction $\mathbf{z} \in \mathbb{R}^2$ (with $\|\mathbf{z}\| = 1$):

$$\mathbf{f}(t) = \sigma \boldsymbol{\xi}(t) + J \sum_i \delta(t - t_i)\mathbf{z}. \tag{32}$$

In this equation, $\{t_i\}$ defines a set of observation times sampled from a Poisson process with rate parameter ν, and $J \in \mathbb{R}_+$ parameterizes the strength of each signal's immediate effect on behavior. To characterize the agent's propensity to move in the cue direction, we decompose its velocity into components aligned

bioRxiv preprint doi: https://doi.org/10.64898/2026.10.02.756268; this version posted October 9, 2026. The copyright holder for this preprint (which was not certified by peer review) is the author/funder, who has granted bioRxiv a license to display the preprint in perpetuity. It is made available under a CC-BY 4.0 International license.

with and orthogonal to $\mathbf{z}$, denoted $v_\parallel(t)$ and $v_\perp(t)$. Let $\bar{v}_\parallel = \lim_{t\to\infty}\langle v_\parallel(t)\rangle$ denote the mean cue-aligned velocity and $\sigma_\perp = \lim_{t\to\infty}\sqrt{\langle v_\perp(t)^2\rangle}$ denote the standard deviation of the cue-orthogonal velocity. The ratio $\frac{\bar{v}_\parallel}{\sigma_\perp}$ is then a natural measure of cue-directedness, somewhat akin to a signal-to-noise ratio.

For a system with closed-loop coupling matrix $\mathbf{A}$, the cue-directedness is given by

$$\frac{\bar{v}_\parallel}{\sigma_\perp} = \frac{J\nu}{\sigma}\frac{\int_0^\infty dt\,\mathcal{H}\left[\mathbf{A},\mathbf{e}_1,\mathbf{e}_1,\tau\right](t)}{\sqrt{\int_0^\infty dt\,\mathcal{H}\left[\mathbf{A},\mathbf{e}_1,\mathbf{e}_1,\tau\right](t)^2}}, \tag{33}$$

where $\mathcal{H}\left[\mathbf{A},\mathbf{e}_1,\mathbf{e}_1,\tau\right]$ is the kernel with which the agent's velocity filters the forcing function (equation 10). This equation consists of two factors. The first factor captures the contribution of the forcing function itself. It is directly proportional to the cue frequency and strength and inversely proportional to the magnitude of the isotropic noise. The second factor captures the closed-loop interactions between the network and behavior. Its numerator corresponds to the DC gain with which the system amplifies signal in the forcing function, while its denominator captures the variance generated by filtering the white noise.

In Figure 6, to explore how the network's dynamics can influence the agent's behavior, we varied the parameters in a network consisting of a single array ($N = 1$) and measured the cue-directedness of the resulting trajectories. More formally,

$$\mathbf{A} = \begin{bmatrix} -\gamma & c \\ b & \hat{w}^{(1)} - 1 \end{bmatrix}, \tag{34}$$

where $\gamma, b, c > 0$, $\hat{w}^{(1)} < 1$, and $\gamma(1-\hat{w}^{(1)}) > bc$ (which ensures stability). We then showed that given the same forcing, the cue-directedness of the resulting path increases as $\hat{w}^{(1)}$ (which sets the neural integration time scale) and bc (which sets the gain of the control loop) increase. Thus, varying both the integration time scale of the network and the gain of the closed loop can enable the agent to exploit this intermittent source of information and stabilize coherent movement in the cue direction.

G Dynamics with velocity nonlinearities

One unrealistic feature of the linear velocity dynamics defined by equation 7 is that the agent's speed is unbounded. This can be addressed by augmenting the velocity dynamics with a saturating nonlinearity that ensures the agent stays below some maximum speed:

$$\tau\frac{d}{dt}\mathbf{v}(t) = -\gamma\mathbf{v}(t) + \mathbf{g}(k_U\hat{\mathbf{u}}^{(1)}(t) + \mathbf{f}(t)). \tag{35}$$

Here $\mathbf{g}: \mathbb{R}^2 \to \mathbb{R}^2$ is a vector-valued function that rescales its input vector by applying a saturating (tanh) nonlinearity to its magnitude while preserving its direction:

$$\mathbf{g}(\mathbf{x}) = \begin{cases} \frac{\tanh(\|\mathbf{x}\|)}{\|\mathbf{x}\|}\mathbf{x} & \mathbf{x} \neq \mathbf{0} \\ \mathbf{0} & \mathbf{x} = \mathbf{0}. \end{cases} \tag{36}$$

Note that this nonlinearity solely alters the dynamics of the velocity vector. The neural dynamics remain linear, following equations 2 and 4. When this speed nonlinearity is introduced, the basic phenomena noted in the preceding sections still hold: increasing the strength of the neural recurrence and the gain of the control loop both increase the straightness of the simulated trajectories.

However, new phenomena also emerge. For example, in the absence of forcing ($\mathbf{f}(t) = \mathbf{0}$), a linear agent (with stable closed-loop couplings) will remain stationary, since its dynamics are dissipative and its behavior is input-driven. The same is true of a nonlinear agent with weak couplings. However, as the nonlinear agent's coupling strengths increase, it can undergo a transition from this quiescent state to an active state in which neural activity and behavior are self-sustaining. This transition can be observed in the single array setup defined by equation 34. To analyze it, we can linearize the dynamics of the

bioRxiv preprint doi: https://doi.org/10.64898/2026.10.02.756268; this version posted October 9, 2026. The copyright holder for this preprint (which was not certified by peer review) is the author/funder, who has granted bioRxiv a license to display the preprint in perpetuity. It is made available under a CC-BY 4.0 International license.

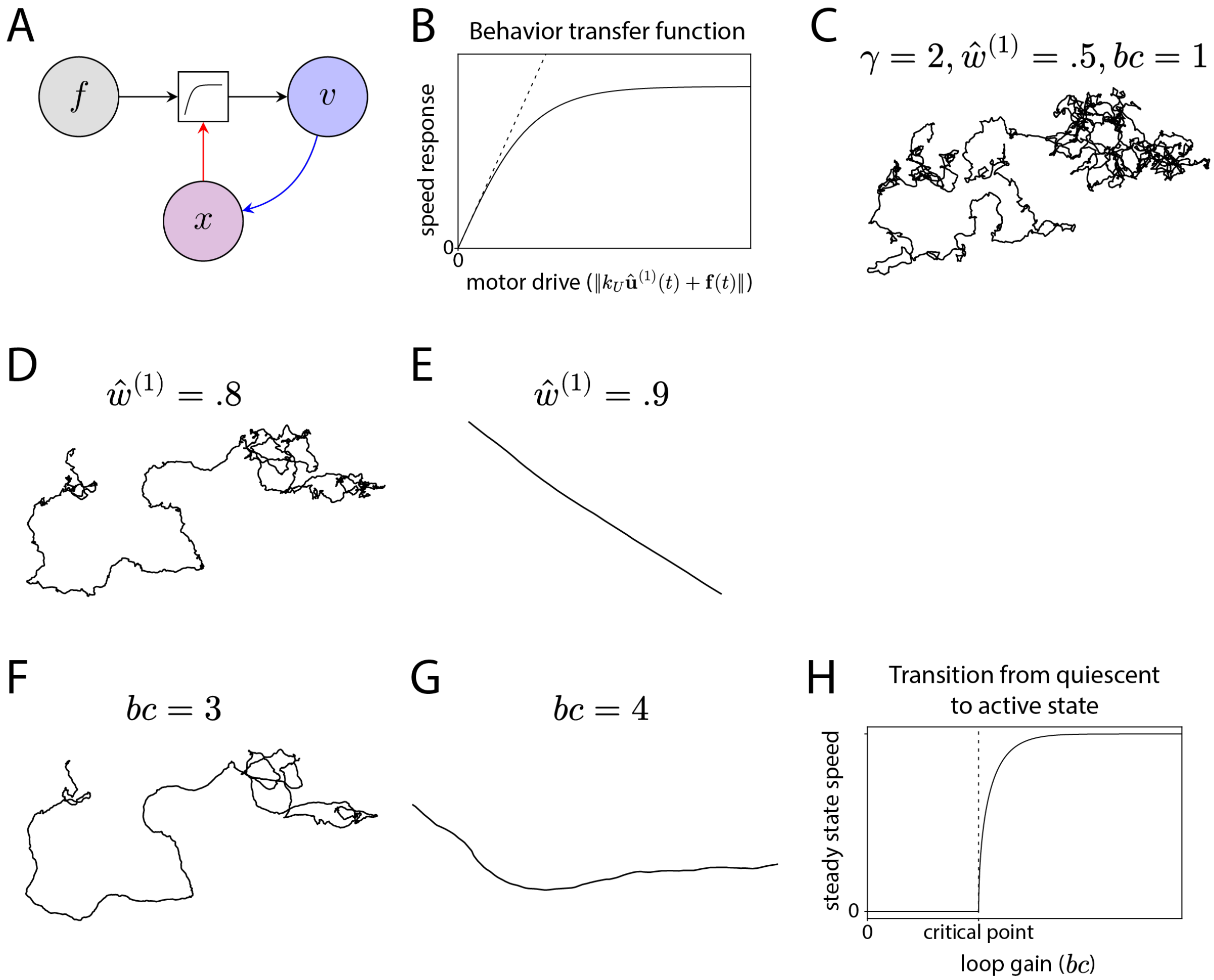

Figure S1: Agent behavior with a speed nonlinearity (A) The motor drive that the agent inherits from the network and forcing function is now mediated by a nonlinear transfer function. (B) This transfer function is a saturating nonlinearity (hyperbolic tangent) that ensures that the agent's speed remains bounded as the magnitude of the motor drive grows. (C) With this nonlinearity, as before, an agent with relatively weak coupling parameters produces a relatively meandering path. (D,E) In response to the same forcing, a network with stronger recurrence can generate a straighter path. (F,G) Increasing the gain of the control loop can also increase path straightness. (H) In the absence of forcing, the network undergoes a transition from a quiescent state lacking movement and neural activity to an active state in which activity is self-sustaining as the loop gain crosses a critical point.

agent's translational speed $V(t)$ and neural amplitude $X_1(t)$ (derived from equations 25 and 27) around the stationary fixed point $V(t) = X_1(t) = 0$:

$$\tau \frac{d}{dt} \begin{bmatrix} V(t) \\ X_1(t) \end{bmatrix} = \begin{bmatrix} -\gamma & c \\ b & \hat{w}^{(1)} - 1 \end{bmatrix} \begin{bmatrix} V(t) \\ X_1(t) \end{bmatrix}. \tag{37}$$

The stability of this fixed point then depends on the leading eigenvalue $\lambda_1(\mathbf{A})$ of the coupling matrix, which is given by

$$\lambda_1(\mathbf{A}) = \frac{\hat{w}^{(1)} - 1 - \gamma}{2} + \sqrt{\left(\frac{\hat{w}^{(1)} - 1 + \gamma}{2}\right)^2 + bc}. \tag{38}$$

When the couplings are weak, this eigenvalue is negative and the fixed point is stable. However, as the eigenvalue crosses $\lambda_1(\mathbf{A}) = 0$, the quiescent state becomes unstable and self-sustaining activity emerges. Notably, this transition can be brought about by increasing the loop gain bc. Thus, even if neural activity and behavior are both dissipative in isolation, they can be sustained by strong sensorimotor feedback between the two. In other words, in this active state with strong feedback, neural activity is persistently maintained by the sensory input that continuously results from the circuit's own motor output.
